# Using photo-elicitation to understand reasons for repeated self-harm: a qualitative study

**DOI:** 10.1186/s12888-018-1681-3

**Published:** 2018-04-11

**Authors:** Amanda J. Edmondson, Cathy Brennan, Allan O. House

**Affiliations:** 1 Centre for Applied Research in Health, School of Human and Health Sciences, University of Huddersfield, Queensgate, Huddersfield, HD1 3DH UK; 2 Institute of Health Sciences, School of Medicine, University of Leeds, 101 Clarendon Rd, Leeds, LS2 9LJ UK

**Keywords:** Self-harm, Self-injury, Photo elicitation, Visual methods, Motive, Reason, Function, Qualitative research, Experience

## Abstract

**Background:**

Reasons for self-harm are not well understood. One of the reasons for this is that first-hand accounts are usually elicited using traditional interview and questionnaire methods. This study aims to explore the acceptability of using an approach (photo-elicitation) that does not rely on solely verbal or written techniques, and to make a preliminary assessment of whether people can usefully employ images to support a discussion about the reasons why they self-harm.

**Method:**

Interviews with eight participants using photo elicitation, a method in which photographs produced by the participant are used as a stimulus and guide within the interview.

**Results:**

Participants responded positively to using images to support a discussion about their self-harm and readily incorporated images in the interview. Four main themes were identified representing negative and positive or adaptive purposes of self-harm: self-harm as a response to distress, self-harm to achieve mastery, self-harm as protective and self-harm as a language or form of communication.

**Conclusions:**

Employing this novel approach was useful in broadening our understanding of self-harm.

## Background

Self-harm is a major public health concern which incurs large costs to healthcare systems [1]. One of its most intractable features is the high prevalence of repeated self-harm, especially in younger people - 15-25% present to the same hospital following a repeat episode within a year [2]. There is also an increased risk of eventual suicide for people who repeatedly self-harm [3].

Most explanations for repeated self-harm focus on deficits such as disordered affect regulation or interpersonal relationship problems (for reviews see Suyemoto [4] Klonsky [5], Edmondson et al. [6]). Current therapeutic approaches typically treat self-harm as a symptom of such underlying pathology and have faced criticism from service users as tending to primarily problematize rather than understand [7]. Focussing on solutions to the problem, mainly through development of interventions with problem solving elements, is a research priority [8]. Yet the evidence so far that such interventions are effective in reducing repetition has not been overwhelming. [9–11]. For example, a Cochrane review of interventions for self-harm in children and adolescents concluded that there is a lack of evidence of effective interventions. Of the relatively few trials of interventions (*n* = 11), most were of low quality [12]. Similar findings were also reported in a Cochrane review of psychosocial interventions for self-harm in adults [13]. Although the number of trials of interventions was greater (*n* = 55) the evidence was reported as “inconclusive” due to the moderate to low quality of the trials, and of those interventions that showed some effectiveness in reducing repetition of self-harm (e.g. cognitive behavioural - based psychotherapy and dialectical behaviour therapy), further trials were needed. It is conceivable therefore that to develop effective interventions which are likely to meet the needs of people who self-harm, a better understanding of why individuals repeatedly self-harm is still required.

A challenge for research in this area is the difficulty experienced by people in verbalising reasons for their self-harm [14–18]. For example, when asked why they have self-harmed people often report feeling unable to put it into words [17]. The act itself has been described as their ‘primary language’ [19].

Despite suggestions that affect can be indescribable and sometimes unknown to the person experiencing it [20], and evidence which shows an association between the trait Alexythymia ‘lacking words for emotion’ and self-harm [21], there is still an assumption that we can articulate our distress effectively [14]. This may explain why first person (verbal) accounts of self-harm often focus on precipitating events *(“I had an argument”*) rather than a more nuanced exploration of its function in the context of these events [22, 23], consequently restricting our understanding.

It has been suggested therefore that future research in self-harm may benefit from an approach that does not rely on purely verbal or written accounts [17]. The value of adopting a visual approach with people who find it difficult to express themselves verbally has been well documented [24–29]. Using participant generated photographs during an interview for example is said to promote expression and communication [30–32]. The visual information evokes a deeper level of consciousness which elicits more of an emotional response than verbal questioning alone, and highlight issues of significance [33]. The benefits of adopting a visual approach when researching sensitive subject areas are also well documented [29, 31–33]. For example, photographs are helpful in introducing difficult subject matter [34]. The photograph(s) can create a sense of distance between the participant and their experience [35] enabling them to opt in/out of direct personal association and talk about an issue more broadly [33].

In this study we therefore undertook an initial exploration using photo elicitation, a method in which photographs or pictures are used as a stimulus or guide in interviews [36]. We aimed to explore the acceptability of using an approach (photo-elicitation) that does not rely on solely verbal or written techniques, and to make a preliminary assessment of whether people can usefully employ images to support a discussion about the reasons why they self-harm.

## Method

### Participants

Working age adults (18 – 65 yrs) admitted to the clinical decision unit or the medical assessment unit of an acute general hospital following a self-harm injury were informed of the research following their self-harm assessment, using information sheets handed out by self-harm team. Service users from a community organisation supporting people who self-harm were also invited to participate through distribution of an information sheet featuring details of the study and contact details of the researcher. Given only a small proportion of people who self-harm attend hospital [37] it was anticipated that capturing experiences from both groups would offer a broader discussion. After initial approaches by staff working in those organisations, those who gave consent to be contacted/expressed an interest were followed up to establish consent and arrange participation. Those people clearly expressing suicidal intent requiring immediate clinical care, requiring translation, or lacking mental capacity were not approached. This was assessed by the self-harm team, where possible.

### Data collection

Participants were asked to take photographs over a two week period of anything that would help them describe their experience of self-harm. Due to ethical concerns and principles of consent, participants were asked to avoid taking pictures of others. All participants were offered the use of a digital camera, although some chose to use their own equipment.

Once the participants had taken their photographs, arrangements were made to meet and discuss the images. Some participants chose to print their images prior to this meeting and brought them along, others selected which images they wanted printing and images were printed by the researcher immediately before the interview. All images were viewed and discussed in A4 colour printed format.

At interview the technique of auto-driving was employed. In this approach a prior topic guide is not used by the researcher but the participant leads or ‘drives’ the interview by choosing which pictures to discuss, in what order and how they talk about the pictures: the researcher adopts the role of ‘active listener’ [38]. Prompts were used to explore thoughts and feelings about presented images and how they represented the participants’ experiences. An ad hoc topic guide was used with one participant who presented without images; this included a discussion around images they might have considered and possible difficulties they encountered. At the end of the interview each participant was informed that the researcher may invite them for a second interview to discuss their experiences further. This was to enable further exploration of any themes following preliminary analyses. Additional consent was obtained from all participants. However, although no further interviews were requested by the researcher, three participants expressed a wish for a further interview. Two of the participants had additional images and issues they wished to discuss, including homosexuality. One participant expressed how after the first interview she had “figured it out”; the uniqueness of the research task appeared to create some initial anxiety. By the end of the first interview she seem reassured and expressed a wish to take more photographs and discuss her experience further. Further interviews were arranged with all three participants two weeks following the first interview though due to varying circumstances (cancellations, intoxication) some interviews took up to six weeks to complete. Interviews were audio-recorded and transcribed verbatim and a copy of the pictures was retained, with the consent of the participants, to be used in the analysis.

To assess the acceptability of using images, the interviewer kept field notes about the tone and conduct of sessions and the use to which images were put during discussions, and reviewed audio recordings for what they said about the use of images as well as what was said about self-harm. Each participant was also asked how they felt about using the method at the end of each interview.

### Analysis

There is currently little guidance on how to analyse combined visual and textual data [39, 40]. Instead, studies reporting visual methods typically employ methods of analysis designed to manage textual data only [30, 41]. In this study however, the aim of the analysis was to capture both the verbal and the visual data. A polytextual thematic analysis developed by Gleeson [39] was therefore undertaken. This method allows the exploration of more than one type of data set whilst working with the assumption that these data sets are linked; meaning is explored by moving back and forward between the data sets rather than seeing them as separate.

Each participant’s transcript and set of images was scrutinised for themes in an iterative process that involved moving back and forward from text to images. Initially, textual excerpts and individual images were scrutinised and extracts of text that were felt to say something were highlighted and qualities within the pictures were noted. The next stage was the creation of explanatory codes (a basic unit of meaning) that could be applied to the textual excerpts and individual images that conveyed the interpreted meaning. Following this stage the data were managed as one source (a list of codes with their associated images and text from an individual); separating the analysis by method of collection was avoided [42]. All coded textual data and images were then reviewed for fittingness by reviewing the text and visual data associated with each code to ensure all the data shared the same meaning. If different extracts of data or images differed in meaning then codes were expanded (or collapsed if different codes had a shared meaning). Deleting codes was avoided in case they became pertinent further down the process of analysis. Codes with similar properties were grouped into tentative themes which were then refined and their boundaries demarcated by further scrutiny of the images and text that had informed the themes. Finally, each theme was defined and named.

This process was repeated for each participant individually and then the themes across the whole data corpus were explored. A framework of tentative master themes was generated from the individual analysis. Tentative master themes were also assessed for fittingness; data were re-examined to explore the ways in which the data were divergent or convergent across individual participants. Where necessary, themes were collapsed or expanded. NVivo, a qualitative data analysis computer software package was used throughout the analysis [43].

Initial analysis was undertaken by the first author and then codes and themes were refined through discussion by all three authors. Using this integrated method of analysis enabled a rigorous and systematic analysis of the textual and visual data from individual experiential accounts of self-harm in the first instance, before concentrating on themes which were common across cases.

As part of the interview, the participants were asked about their experience of photo-elicitation. During analysis, the authors held extensive discussions on the nature of the images presented and on the types of images that were not present. Notes from these discussions and from the participants’ responses were used to make an assessment of the role of images in the interviewing process, as reported elsewhere [44, 45] .

## Results

Consent to be contacted by a researcher was obtained from 28 people; however contact could only be established with 20 people. Of these, thirteen consented to participate in the research; two declined due to housing difficulties; two declined due to low mood; and three people declined due to feeling unable to discuss their self-harm at the time of the research.

Of these thirteen, eight people provided data; three withdrew consent (reasons included further inpatient treatment and issues with probation) and two did not respond to attempts to contact them.

### The participants

Eight adults, two males and six females, aged between 21 and 65 participated in the study. A total of eleven interviews lasting between 40 min and two hours were carried out and 143 photographs were collected (mean number 18 images; range 0–66). One participant, presented without images and an ad hoc topic guide was used.

Five of the participants reported a long history of self-harm using varied methods. Of those, one participant also reported a long history of an eating disorder and addiction to alcohol. Three participants, reported self-poisoning only during a particularly difficult period of their life.

Participants also reported having suffered varied mental health problems. Self-reported diagnoses included schizophrenia, drug induced psychosis, depression, alcoholism, bulimia, and dissociative identity disorder.

### Observations about the use of images

#### Number and variety of images

In addition to the number of images presented by participants we noted their variety*.* For example, familial and intimate relationships as well as close friendships were represented through images, as were interior and outdoor spaces. The range and number of images facilitated detailed discussions about self-harm in terms of specific triggers, methods of harm and functions; they also elicited discussions of the significance of people and place.

We were struck by the absence of some images that are frequently present on internet sites and accessible via social media. We were shown no pictures at all of actual injuries. This might reflect a difference in the personal uses to which people will put images, from those which lead others to place images in the public domain. Since the purpose of the study was to explore the reasons why people self-harm, participants may have chosen images that would enable a discussion of the purpose it served for them, rather than the end result. Alternatively our proscription of pictures of identifiable individuals may have been interpreted as an implicit instruction that only certain more impersonal images were acceptable.

#### Use of images in interviews

The images were readily incorporated into the interviews and there were many occasions where seemingly mundane images, such as a road works sign, unveiled complex narratives relating to self-harm; the interaction between image and narrative was important in understanding what was being communicated.

**Fig. 1:**
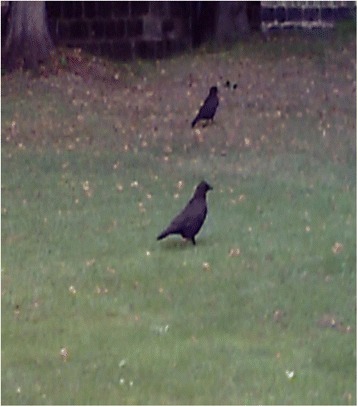
The birds

Both males in this study, participants 3 and 8, talked about their experiences in a very visual way. Participant 3 in particular presented pictures to represent the contents of his flashbacks; four out of five images represented traumatic experience which he found difficult to verbalise. One of the images captured two birds, see Fig. 1. He discussed how he wanted to capture an image of a heron; he described a fear of herons and how the sight of one would trigger an act of self-harm. He went onto describe how the image (of a replacement bird) represented a very abusive relationship with his mother. His other images also featured images to represent different abusers and places of abuse.

#### Engagement with use of imagery

Some participants seemed energised by the task of producing images; participant 2 had arranged some of her images into a collage for the interview. Participant 6 described how the use of visuals had allowed her to express her experiences in a way others could understand:

*“You could translate into something that somebody else could understand like, like the volcano, how would you explain that? Whereas you show them the volcano it’s more obvious than words. I suppose people will understand volcanos.” (Participant 6, p.14 line 614)*

We noted that some people seemed more familiar with, or receptive to, the idea of taking photographs than others. Only one participant (participant 5) produced no pictures despite consenting. Some respondents seemed more innately “visual” in their thinking. Participant 1 for instance noted how a change in her emotional status also saw a change in her physical appearance:

*“every time I’ve done it [overdosed], I’ve dyed my hair…it’s a bit weird that like every time I’ve done it I’ve kind of tried to change my appearance as well” (Participant 1, p.12 line 483 interview 1)*

#### A positive experience

Participants reported having enjoyed using photographs to describe their experience of self-harm. They described it as “helpful”, “a good thing” and “interesting”. Participant 1 described how the photograph served as a tool which helped her begin and continue talking about her experience:

*“it’s quite a good thing because if you were just to say come in and talk about it I wouldn’t know where to start and its good like, it’s a talking point, like the picture you can say I’ve taken this because and then it leads, like the picture of my dog, it’s a picture of a dog but it causes this and that you know what I mean” (Participant 1, p.11 line 511, interview 1)*

Participants reported feeling able to capture images which represented their personal experience of self-harm. Each participant led their interview and for the most part they seemed at ease throughout. Having prepared for the interview by taking and choosing images in advance, (and perhaps considering what they wished to discuss in relation to each image prior to the interview), served to facilitate the interview.

#### A challenging experience

Almost certainly, capturing images to represent experience of self-harm was more of a challenge for some than others. For some the biggest challenge seemed to be getting started and thinking about what they wanted to capture, and then finding the image (e.g. a heron). Most of the participants described the process as something which gathered momentum. Finding images to express emotional states was described as difficult by participant 1 but then she came up with her own solution:

*“I don’t understand how I can take a picture of anger, like I guess I could take a picture of something that causes the anger which I did” (Participant 1, p.12, line 526, interview 1)*

Some participants apologised for their images and seemed to lack confidence when showing them. Some seemed embarrassed and perhaps felt under pressure to produce images of great interest, which in turn might have inhibited their ability to express their experience of self-harm.

### Explanations for self-harm

#### A response to distress

The analysis identified a number of themes that support commonly recognised explanations for self-harm such as: self-harm as punishment, self-harm as a relief from pain (affect regulation) and self-harm as a counter to loneliness.

Loneliness was a common theme across all participants with discussion often centred on the scarcity of human contact. Many of the images presented seemed to depict loneliness with a predominance of bare rooms sometimes with single cups on a table.

Self-harm as a form of punishment was also a common theme. Participant 4 presented a number of images of barbed wire and one of a rusty medieval looking arrow tip. She described how her self-harm was an act of punishment for bad thoughts and deeds as well as things not done:

*“…it was punishment but it was kind of good punishment because it hurt but I got a satisfaction out of it as well, and it served a purpose so it was, it’s always been a very contradictory thing of pain only being soothed by more pain.” (Participant 4, p.12 line 656)*

There was much discussion of self-harm as a way to manage emotions when things got too much. For example, one of the images presented by participant 6 was of a closed door and she described her self-harm as a way to take a pause on her life, not in the sense of contemplating suicide, but temporarily taking a respite when things got too difficult.

*“Yeah just sick of dealing with all the shit cos it’s one thing after another after another sometimes you think just let me step off for a bit and I can’t deal with anymore shit thrown my way” (Participant 6, p.11 line 461)*

**Fig. 2:**
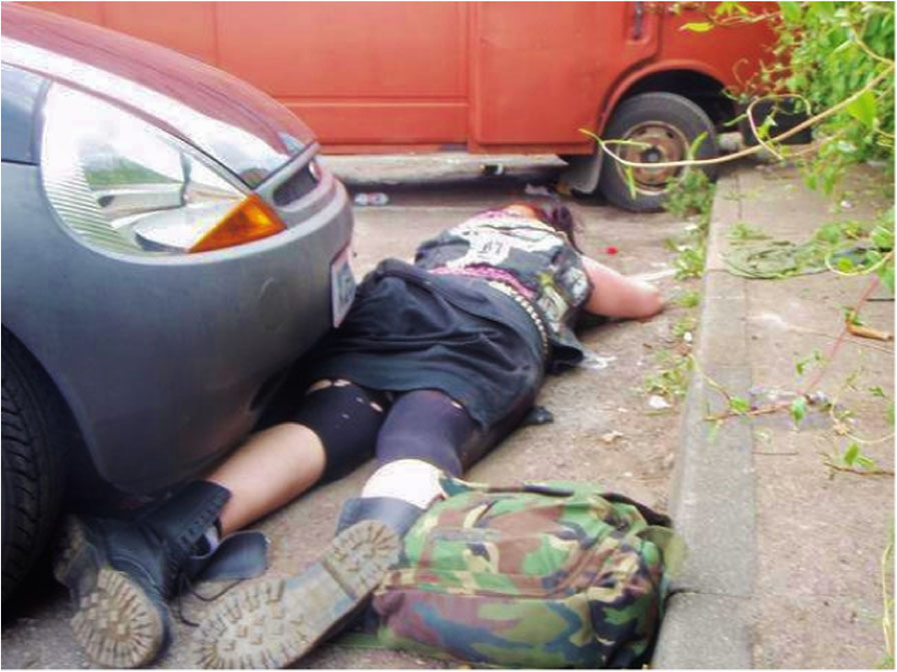
At rest

Participant 1 described how self-harm was a way to help her sleep and reach a sense of calm when things became overwhelming and she presented a picture of her lying in front of a car seemingly “at rest” see Fig. 2.

It was an interesting choice of picture in that it could be interpreted as quite the opposite, for example it might suggest vulnerability, risk and disorder. This interaction between the image and narrative was important to really understand what was being communicated. Showing images which depicted the opposite of what was said, and how they preferred to be perceived was particularly notable in the narratives of participants 1 and 3. They both discussed a need to present a hardy persona. Participant 1 described how she preferred her friends think that she is fine, rather than “a mental bitch”. Participant 4 similarly presented a number of images which depicted a desire to appear strong, yet on the inside she felt completely broken. The discussion of images helped reveal both their internal and external selves and describe how self-harm can be protective in that it allows the internal self to remain hidden, whilst offering a sense of mastery (being in control of the perception of others).

#### Self-harm as protective:

Many of the participants reported experiencing adverse events throughout their lives, such as sexual abuse, death of significant others and bullying. When discussing these experiences a common thread throughout the narratives was the apparent lack of protective factors within their life. This was usually expressed through feelings of vulnerability, loneliness and a perceived lack of care from others.

**Fig. 3:**
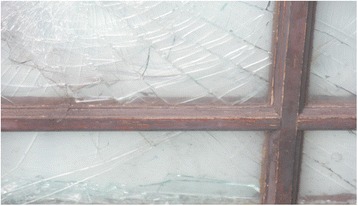
Broken glass

A sense of vulnerability was visually represented by participant 4 through images of glass sheets that had been shattered but had not yet fallen apart. Her narrative picked up this theme by describing how she always felt she was shattered on the inside but never actually broken. Whilst  reflecting on the images, she described how her experiences had left her internally damaged but that she felt she needed to project an external sense of herself as someone who would never break; they depicted something brutally damaged but still intact, see Fig. 3. Participant 4 also presented an image of a brick wall which could both depict external strength but also act as a barrier to conceal her inner turmoil:

*“Stay upright, stay together and not cross those boundaries so people would find out what was going on because that was something that I couldn’t do so I had to internalise it.” (Participant 4, p.4 line 136)*

#### A sense of mastery

**Fig. 4:**
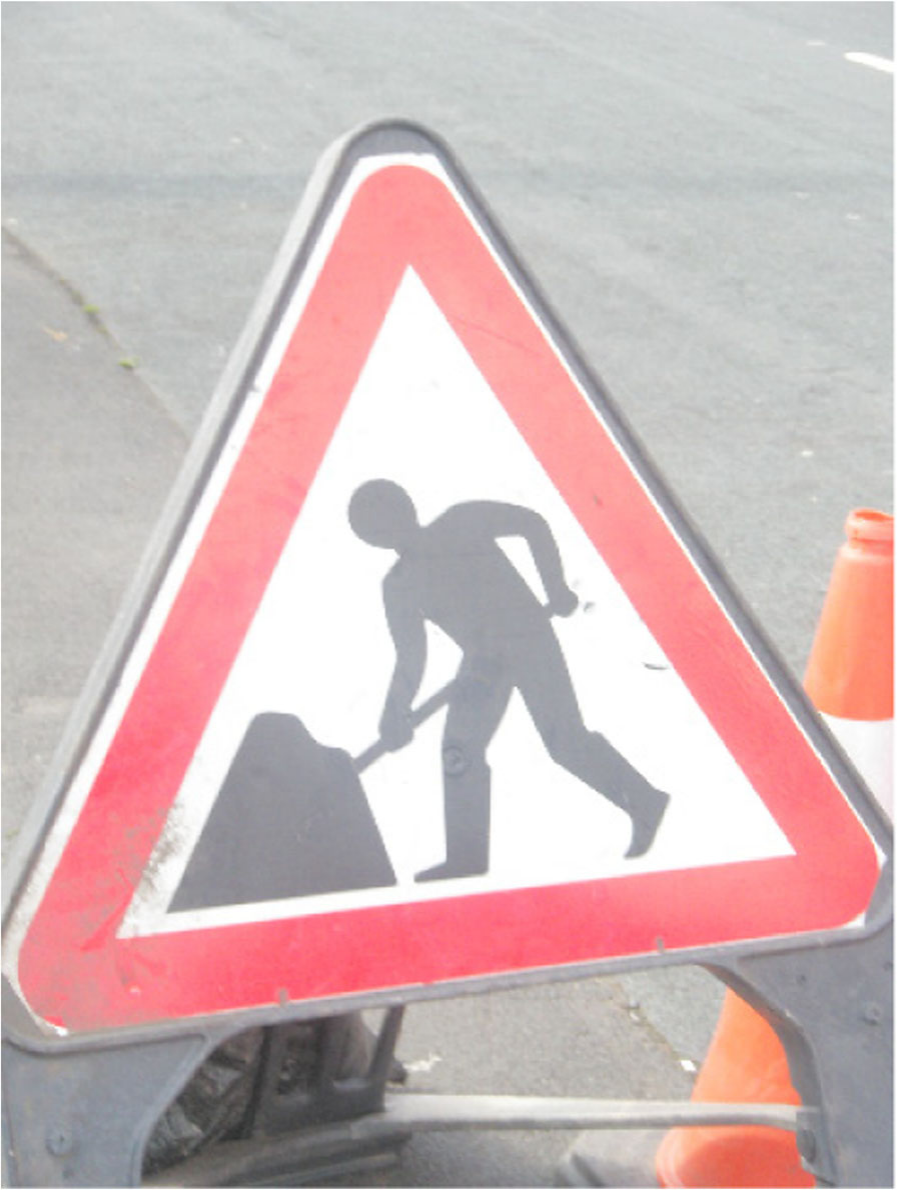
Roadworks

The theme of self-harm as a sense of mastery captures how feelings of control (or a lack thereof) were experienced in different and complex ways as both an antecedent to and a function of self-harm. Fundamentally control was something participants felt they lacked. It was described in terms of a generalised feeling of lack of agency and also as a result of being controlled by another or others. Participant 4, a young woman who had a long history of self-harm, discussed a dislike of her life of ‘chaos’ and disorder and central to her account was the value of being able to reduce her sense of ‘chaos’ through the act of self-harm. For example, to represent her chaotic life she chose images of winding paths and dark stairwells which for her captured a sense of uncertainty. To counter this she presented an image of a road sign to indicate roadworks ahead. She described the roadworks sign, which for her represented the act of self-harm, as an indication that she could now sense what was happening ahead and that there was the possibility of repairs to the chaos, see Fig. 4.

**Fig. 5:**
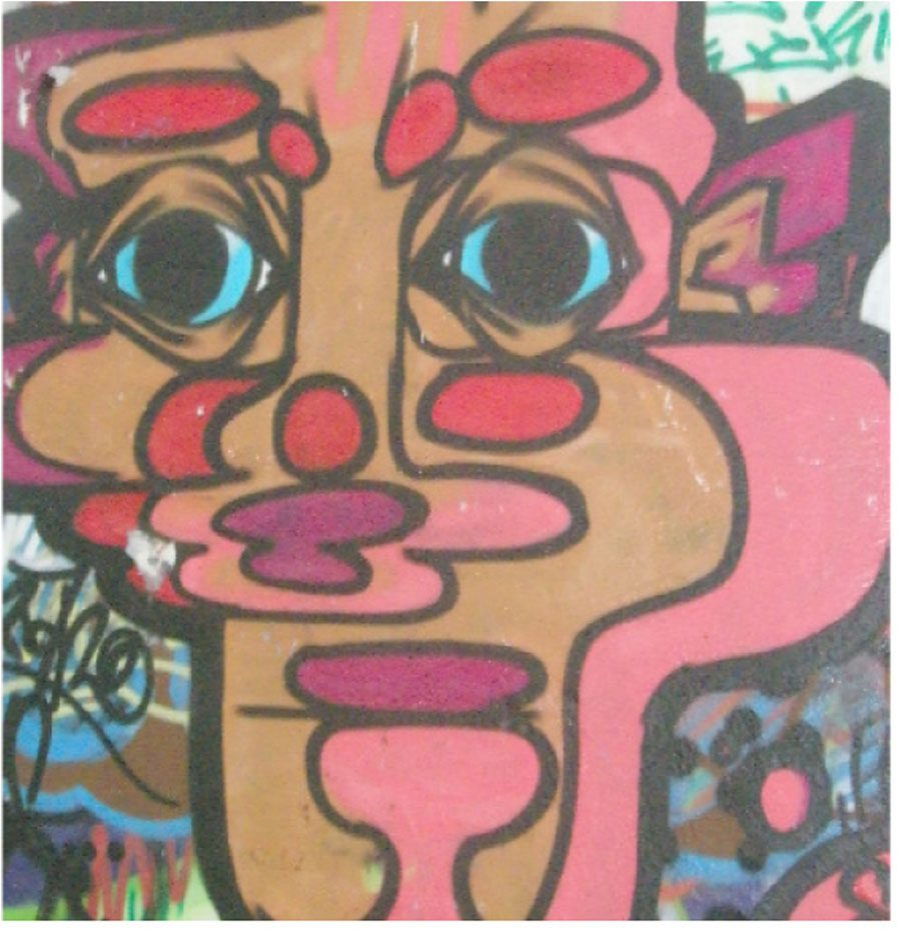
An evil inside me

Some participants reported feeling as though their lives were heavily controlled and manipulated; their sense of control was lacking as they felt controlled by someone or something else. Participant 4 described an ‘evil’ inside her and self-harm was a way to exert some control over this, see Fig. 5

*“I had so many times where I was like, I need to cut because I need to, I can’t stop the evil, I can’t stop it taking over and putting all these pictures in my head and I thought ultimately it was going completely take over my personality and I was going do all these horrible pictures that I was seeing in my head to other people. So I needed to slow it down so it was very logical of ok how do I slow down something that’s in my blood would be to cut cos I’m releasing the blood therefore I can slow down the evil” (Participant 4, p.15 line 671)*

This sense of control the participants gained through self-harm was also evident when participant 4 talked about being her own master of hurt; self-harm gave her control and although she was able to reflect that this might seem irrational, she gained positive benefits from this thought.

#### Self-harm and communication

Across participants’ accounts of self-harm the theme of communication was very apparent. Many of the participants discussed images representative of communication difficulties.

**Fig. 6:**
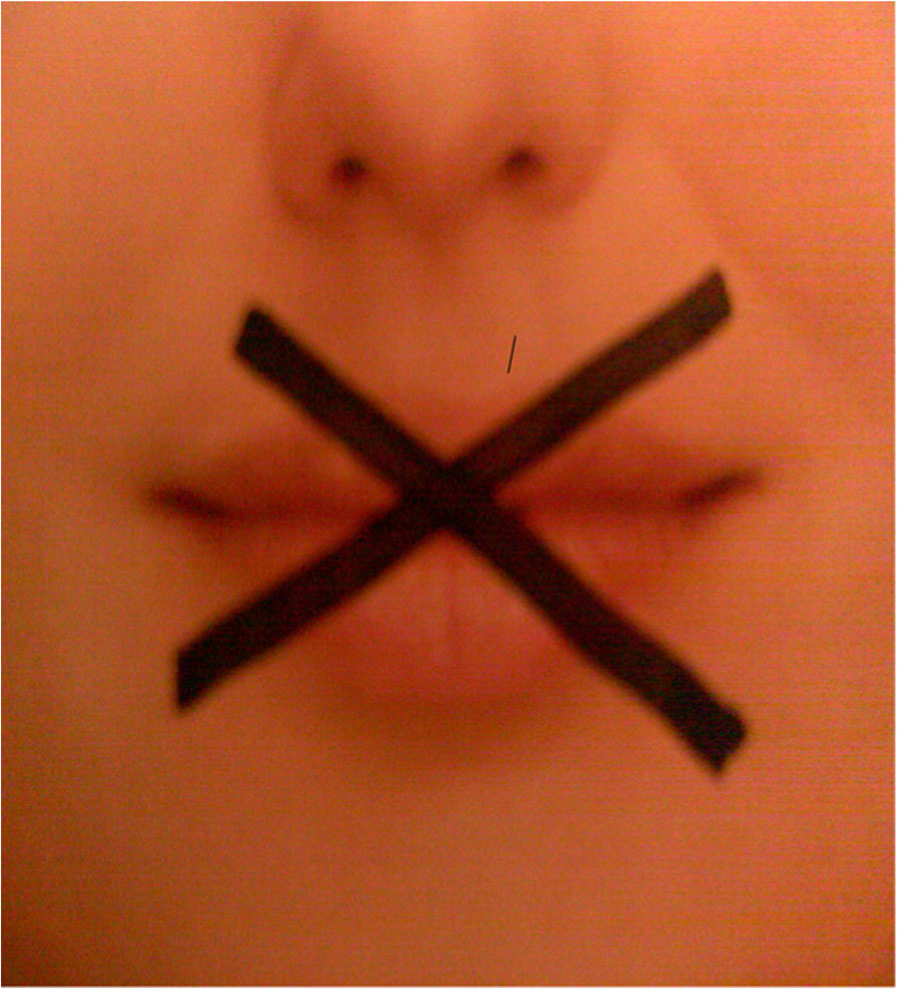
Difficult to communicate

For example, participant 6 presented an image of a women’s mouth crossed shut with black strips, see Fig. 6. She discussed how she had tried to talk to people about her self-harm in the past but she felt that they “just didn’t get it”.

Being unable to communicate satisfactorily was expressed in several ways. First, the use of words was sometimes described as inappropriate and ineffective, some difficult and sensitive experiences were felt to be ‘beyond words’. Some participants expressed an inability and reluctance to express themselves through words because of negative experiences or a lack of experience in using words to communicate issues of a sensitive nature.

*“Actions speak louder than words don’t they” (Participant 7, p.3 line 112)*

*“I don’t use my words, so the pressure builds then I, I cut and that’s how I deal with that” (Participant 4, p.9 line 415)*

Some participants did describe their self-harm as a form of communication to others of inner turmoil or pain that they either could not find the words for or did not think the words were understood.

However, in this theme self-harm was more nuanced than simply social communication, seeking attention or help. The act or injury was often seen as a signifier to the self that things were different, or might be different going forward.

For example, participant 3 used a grammar metaphor to describe his self-harm. In this sense, the communication was not with others but as a message to himself:

*“Self-harm is like a full stop, like punctuation, it’s punctuation, it’s a sort of punctuation to moods or emotions or to a series of memories” (Participant 3, p. 8 line 359)*

There was a recognition from some of the participants that using self-harm as communication could be problematic; participant 7 described how she had been criticised by others and at this point in the interview she presented an image of her notebook which symbolised a shift in the way she now expresses herself.

*“my notebook and erm since like the erm self-harming happened and stuff I’ve started like writing like my negative thoughts and feelings down it’s like, its more about how I’m dealing with it now…I find it really helpful to just write things down that I’d want to say to him like angry feelings and how he made me feel so that I won’t say them to him or to anybody else or I’ll like get back in that bad place” (Participant 7, p.12 line 601)*

## Discussion

The aim of this article was to explore whether using a novel method to elicit reasons for self-harm would help participants talk about their experiences and therefore provide a more nuanced understanding of why people self-harm.

### Utility of the method

One of the main purposes of adopting a visual methods study with people who self-harm was to enable them to feel as though they were in control of the research process and offer them a different form of expression. It has been interesting to see how those key features were discussed by the participants as their functions of self-harm. Perhaps through enabling a different form of expression (from conventional methods), yet similar to their chosen form of expression (self-harm), participants felt more able to express and communicate their experience of self-harm. For example, others have suggested that people draw upon visual images during times of psychological distress [46–48] . Holmes et al. [47] and Hales et al. [46] both reported how participants experienced detailed mental imagery about future suicide attempts, which they termed ‘flash forwards’. They suggested ‘flashforward’ imagery warrants further investigation for formal universal clinical assessment procedures.

Moreover, the use of metaphorical and figurative speech featured widely throughout most of the participants’ accounts which would suggest a propensity to describe experiences of distress through imagery.

### Explaining self-harm

#### Self-harm as a response to distress and to punish oneself

As described in reviews by Suyemoto [4], Klonsky [49] and more recently Edmondson et al. [6], evidence of self-harm serving to regulate affect (to get relief from negative feelings) and punish oneself (show anger toward oneself to self-soothe) were also found in this study. Both functions are particularly well documented in the literature; a systematic review of self-reported reasons for non-suicidal self-injury, which included accounts of 29,350 participants, found the majority of studies (49/152 articles, 98%) reported evidence of affect regulation as a function of self-harm. Over half (92/152, 60%) reported punishment as a function of self-harm [6].

#### Self-harm and sense of mastery

Self-harm was described as a behaviour through which feelings of control, empowerment and ownership could be sought. The subject of control has been well documented and the evidence suggests that self-harm offers a feeling of control through feeling able to rid oneself of or reduce unpleasant affective states, commonly referred to as affect regulation [4, 5, 18, 50–53]. The findings from this study and others however have shown how control can be gained through the behaviour in and of itself, for example through controlling the level of pain, depth of cut and the amount of blood [18, 49, 51–55]. Moreover, our participants and others described a sense of ownership over their behaviour, remarks such as “*it’s mine*”, “*there are certain things they can’t have and that’s [self-harm] one of them*”. Such statements suggest there are positive experiences to be gained through self-harm. These sorts of experiences resonated with those participant responses in Shearer’s [55], Demming’s [51] and Brooke and Horn’s [53] studies who all studied women’s reflections of their self-harm. One participant in Demming’s [51] study described her self-harm as something that belonged to her, that she controlled and only she could stop it. Shearer [55] on the other hand included the statement “*to do something I have control over and no one else can control*” within a questionnaire and the item was ranked one of the top three functions by 22% of participants.

#### Self-harm as protective

Experiences of sexual abuse, death of significant others and mental health problems were common in our participants as they are among most populations where repeated self-harm is found. In response to such experiences self-harm seemed to function as a protection. Usually protective factors - “predictors of positive outcomes among people at risk for developing problems as a result of adverse life events or experiences” [56] - are thought of as a supportive network of family or friends [57]. How can self-harm act as a substitute?

The protective properties of self-harm were expressed in different ways, again, some of which resonated with functions such as affect regulation [4, 5] and anti-suicide (where a person self-harms to avoid suicide) [4, 5].

The experiences captured in this study however, again, seemed encompassing of something more than affect regulation and anti-suicide. For example, self-harm was described as a behaviour through which feelings of protection and preservation could be sought. Similar descriptors have been reported in other studies and articles, for example, metaphorical statements such as “*it’s my life raft…a sort of safety shield,*” [58].

Collectively these findings support the idea that self-harm serves to regulate feelings of distress, but they also suggest that self-harm can be adaptive and can offer something positive beyond the elimination of distress. For a more detailed discussion about positive and adaptive functions of self-harm see [6].

#### Self-harm and communication

The theme of communication was very apparent throughout the personal accounts of self-harm. Klonsky [5] and Suyemoto [4] both described how people use self-harm as a way of interacting with their environment. Klonsky [5] refers to the ‘interpersonal influence’ model to describe how people use self-harm to influence or manipulate people in their environment. Suyemoto [4] refers to the environmental model to describe how self-harm creates environmental responses that are reinforcing.

The four function model [59, 60] proposes that people use self-harm as a language to serve a social function that relates to items such as “to get other people to act differently or change”, “to try and get a reaction from someone, even if it’s negative”, and “to make others angry”. Nock [61] also compared self-harm as a language to somatoform behaviours, whereby physical symptoms are presented as an alternative means to communicating psychological distress.

Although the environmental model does include how self-harm can be used to express the inexpressible, which might seem related to the idea of using self-harm as a language, these models do not satisfactorily explain how people used self-harm as a language in this study.

Messages were ‘written on the body’ in the same way Adshead [14] described, through the act of self-harm and this was used to do the talking that participants felt unable to for the reasons discussed – not just to seek help or for an immediate social function but in a more personal way – described in other research as a form of remembrance, like creating physical reminders of important events [49].

### Strengths and limitations

To the best of our knowledge this is the first study to use photo elicitation to explore reasons for self-harm and to an extent, this method encouraged participants’ to use images in the same way they use their body, as a way of expression. This visual way of expression allowed the researcher to ‘see’ what was often hidden and private but in a controlled way. All of the images were generated by the participant which facilitated a safer, more controlled disclosure. For some, it was reported as the first time they had ever spoken in such an honest and detailed way about their self-harm. The study yielded rich, distinct, visual and verbal data (over ten hours of interview, featuring 143 images). However, given the small sample size (*n* = 8) we cannot be confident of saturation or transferability to the population as a whole. Similarly, although some participants were interviewed on more than one occasion, further interviews with all participants following a preliminary analysis would have allowed further exploration of some of the more novel themes (i.e. protection and mastery), and a more detailed discussion of all the images in cases where excessive numbers of images were generated. More emphasis on the process of taking images could also have been discussed in subsequent interviews to enable a more comprehensive critique of the method. Future research into self-harm should consider the strengths and limitations of certain research approaches to ensure a more complete understanding of the reasons why people self-harm, to help develop interventions which are likely to meet the needs of people who self-harm.

## Conclusions

Taking pictures is familiar; personal lives now seem perpetually pictorially documented through social media. However, on reflection taking pictures to represent difficult experiences is not as familiar and requires more thought. Using pictures to represent experiences of self-harm required effort, abstract thinking and reflexivity [30] which some people struggled with more than others. For some this approach was possibly perceived as a measure of their ability – observations made in a different context by Mannay [62], Packard [63] and Frith and Harcourt [36].

These characteristics of photo-elicitation – the degree to which it requires concerted and unfamiliar effort from the informant, and yet offers control over the content of what is discussed – are differences from traditional language-based methods and arise directly from its use of images. We found that most participants responded positively, produced multiple appropriate images and discussed them actively – revealing aspects of their reasons for self-harm that are less well documented. These observations suggest that photo-elicitation has potential as a method for clinical or research use in self-harm work and further evaluation is justified.
